# Shengjiang San alleviated sepsis-induced lung injury through its bidirectional regulatory effect

**DOI:** 10.1186/s13020-023-00744-6

**Published:** 2023-04-17

**Authors:** Shifan Yan, Yu Jiang, Ting Yu, Changmiao Hou, Wen Xiao, Jing Xu, Huili Wen, Jingjing Wang, Shutong Li, Fang Chen, Shentang Li, Xiehong Liu, Hao Tan, Lianhong Zou, Yanjuan Liu, Yimin Zhu

**Affiliations:** 1 Department of Emergency, Institute of Emergency Medicine, Hunan Provincial People’s Hospital (The First Affiliated Hospital of Hunan Normal University), 69 Jiefang Western Road, Changsha, 410000 Hunan People’s Republic of China; 2 Hunan Provincial Key Laboratory of Emergency and Critical Care Metabonomics, Changsha, Hunan China; 3 Hunan University of Chinese Medicine, Changsha, Hunan China; 4 Department of Pediatrics, Third Xiangya Hospital of Central South University, Changsha, Hunan China; 5 Jiangxi University of Chinese Medicine, Nanchang, Jiangxi China

**Keywords:** Sepsis, Shengjiang San, Network pharmacology, Pyroptosis, Lung injury

## Abstract

**Background:**

Sepsis is a life-threatening organ dysfunction caused by dysregulated host responses to infection, for which effective therapeutic strategies are still absent. Shengjiang San (SJS), a well-known Traditional Chinese Medicine formula, has been widely used clinically. However, its role in sepsis-induced lung injury remains unclear.

**Methods:**

To explore its specific mechanism, we firstly established a sepsis animal model using cecal ligation and puncture (CLP) and treated MH-S cells with LPS plus ATP. Then, UPLC/Q-TOF–MS/MS was utilized to identify its active ingredients. Network pharmacology analysis was performed to uncover the potential mechanism. HE staining and biochemical analysis were conducted to validate its therapeutic effect. ELISA was applied to detect the release of pro-inflammatory and anti-inflammatory cytokines. Western blot was utilized to detect the protein levels of GSDMD, NLRP3, P65, ASC and caspase-1.

**Results:**

SJS could dramatically increase the survival rate of sepsis. In addition, it is able to inhibit the pro-inflammatory cytokines release at day 1 post CLP while promote their production at day 7, indicating SJS could attenuate uncontrolled inflammatory response in the early stage and improve immunosuppression in the late phase. Network pharmacology analysis showed that pyroptosis is the crucial action SJS exerted in the protection of sepsis-induced lung injury. Western blot data implicated SJS could attenuate pyroptosis in early sepsis while enhance in the late phase.

**Conclusions:**

SJS acted to alleviate sepsis-induced lung injury through its bidirectional regulatory effect.

**Supplementary Information:**

The online version contains supplementary material available at 10.1186/s13020-023-00744-6.

## Introduction

Sepsis is a biphasic disease characterized by an initial uncontrolled inflammatory followed by a prolonged immunosuppression phase [1]. The immune response during the sepsis progression is reported to be quite complex. Several studies demonstrated that an imbalance between pro-inflammatory and anti-inflammatory cytokines is the major characteristics [2]. To date, the therapeutic effects using anti-inflammatory treatment approaches or immunoadjuvant therapies remain less than satisfactory due to the absence of clear-cut borderlines between the early stage and late phase [3, 4]. Therefore, exploring novel drugs acted to maintain the immune response balance throughout the whole disease progression is the crucial strategies to improve the disease prognosis.

Pyroptosis, a programmed cell death, is majorly triggered by the activation of NLRP3 inflammasome complex [5]. Various stimuli could induce the formation of NLRP3 inflammasome complex and recruit the ASC protein as well as pro-casapse-1, thus resulting in the activation of caspase-1 and the cleavage of GSDMD [6, 7]. Caspase-1 activation could further cleavage pro-IL-1β and pro-IL-18 into their active forms [8]. GSDMD acted to pore in the plasma membrane and increased the release of IL-1β and IL-18 [9]. Pyroptosis is reported to be a double-edged sword during the sepsis progression. In the early stage, pyroptosis-mediated IL-1β and IL-18 overproduction triggered cytokines storm and lead to multiple organ injury [10]. Subsequently, pyroptosis was decreased and immunosuppression was seen. Meanwhile, pro-inflammatory response was observed to be attenuated and patients were generally more susceptible to secondary infection [11]. Although pyroptosis has a negative effect on early sepsis, it has a positive influence on the immunosuppression phase because of its potent pro-inflammatory activity [9]. Therefore, exploring novel drugs to bidirectional regulate the pyroptosis perhaps the potential therapeutic strategies for the treatment of sepsis.

Shengjiang San (SJS), a widely used Traditional Chinese Medicine formula, functioned to clear heat and detoxify. It is composed of Rheum officinale, Rhizoma curcumae longae, White muscardine silkworm and Periostracum cicada at ratio of 4:3:2:1 [12]. Modern phaemacological studies demonstrated that SJS executed various biological activities, such as antibacterial, anti-inflammatory, antioxidant and immune boosting [13]. It is reported to inhibit the activation of NF-kB signaling pathways and alleviate systemic inflammatory response syndrome, indicating SJS may play a protective role in the early stage of sepsis [14]. Additionally, several studies also demonstrated it acted to enhance immune response via regulating CD4^+^ and CD8^+^T cells to balance Th1/Th2 [15]. Our previous research revealed it could maintain immune homeostasis by modulating the proportion of CD4^+^PD-1^+^ T cells and the ratio of CD4/CD8. Therefore, we speculated that SJS perhaps play a protective role during the whole disease progression of sepsis. However, it is still unclear whether SJS could bidirectional regulate pyroptosis.

In the present work, we sought to investigate the therapeutic effects of SJS during the whole sepsis progression. We also determined whether SJS maintains immune homeostasis by bidirectional regulating pyroptosis.

## Materials and methods

### SJS preparation

The SJS including 180 g Rheum officinale (No.210429), 135 g Rhizoma curcumae longae (No.201023), 90 g White muscardine silkworm (No.210228) and 45 g Periostracum cicada (No.210409) was obtained from the Affiliated Hospital of Jiangxi University of Traditional Chinese Medicine. All herbs are in compliance with the Chinese Pharmacopoeia. They were soaked for 30 min and decocted twice with an eightfold volume of distilled water for 1 h. The decoction was obtained by filtration through 8 layers of gauze. The filter residue was repeated extracted for one time with a sixfold volume of distilled water and filtered the decoction. Subsequently, concentrated the filter at the temperature of 60 °C to obtain the concentrated one. Then the concentrated solution was frozen at – 20 °C overnight and dried in a vacuum freeze drier for 48 h. Eventually, 36 g of powder was acquired, with a yield of 8%. Conversion rates were calculated in humans and mice based on clinical dose and pharmacological experimental methods (SJS human clinical dose: 45 g/day, adult weight 60 kg, the dosage of mice were: 45 g ÷ 60 kg × 9.1 ≈ 6.75 g/kg) [16].

### Animals

C57BL/6 male mice aged 6–8 weeks were purchased from the Saike Jingda experimental animal Co. Ltd (Changsha, Hunan, China). All mice were housed in a specific pathogen-free environment at 25 °C with a 12:12 h day/night cycle and raised in separate cages. The animals applied and the protocols operated in our study were approved by the Medical Ethics Committee of Hunan Provincial People's Hospital (Grant No.2021-100).

### Sepsis animal model established and SJS treatment

All animals were underwent of a fast for 8 h and a deprivation of water for 4 h, followed by the surgery of a cecal ligation puncture (CLP). During the process of the surgery, the animals were anesthetized with 2% isoflurane and anesthesia maintained throughout the operation. Subsequently, we made a 1 cm incision along the abdominal and exposed the cecum under the condition of anesthesia. A vertical ligation of 4–0 silk thread was performed on approximately 75% of the cecum after exposure to ensure uniform distribution of the internal contents and a needle of 21 gauge was utilized for double puncture [17]. In the following experiments, animals were randomly divided into five groups: (1) sham group: mice have only freed the cecum but without ligation and perforation; (2) CLP group: mice were intragastric administration of normal saline after the surgery of CLP; (3) low-dose group: a dose of 3.375 g/kg SJS were intragastric administration after CLP surgery; (4) equivalent-dose group: a dose of 6.75 g/kg SJS were intragastric administration after CLP surgery; (5) high-dose group: a dose of 13.5 g/kg SJS were intragastric administration after CLP surgery. SJS or normal saline was given 2 h after the surgery and continuous administrated twice per day for 1 day or 7 days.

### Serum collection

At 1 and 7 days after the surgery, mice were anesthetized with 2% isoflurane and serum was collected. The collected serum stored at − 80 °C for biochemical analysis and inflammatory cytokines detection.

### HE staining

The liver, lung and kidney tissues from the mice was quickly collected and fixed with 4% paraformaldehyde for 24 h, according to the standard protocol. Subsequently, the 5 mm thick sections were embedded into the paraffin. Then, Hematoxylin–eosin (HE) staining was applied to examine the change of morphology in liver, lung and kidney tissues and Immunofluorescence staining was used to detect the P65 level in lung tissue.

### Immunofluorescence staining

Immunofluorescence staining was applied to detect the p65 phosphorylation level in MH-S cells. Briefly, MH-S cells were seeded into 12-well plates with cell climbing slice at a density of 2 × 10^4^ cells/well and cultured in 1640 complete culture medium supplemented with 10% FBS, followed by the treatment with different concentrations of LPS together with ATP. After that, the cells were fixed with 4% paraformldehyde for 10 min and broken with 5% Triton- × 100 for 20 min. Then, washed with PBS for 3 times and blocked with 5% BSA for 1 h. Subsequently, primary antibody against p65 was added (1:200 dilution) at 4 °C overnight and goat anti-rabbit IgG (dilution 1:250) served as secondary antibody. The cells were visualized using a fluorescent microscopy.

### UPLC/Q-TOF–MS/MS analysis

The active ingredients of SJS was identified by Fuda Analytical Testing Group using UPLC/Q-TOF–MS/MS analysis. Firstly, 100 mg of samples was placed in a 15 mL centrifuge tube, followed by the addition of 10 mL 50% aqueous methanol solution and sonication for 30 min. 1 mL of the supernatant was transferred to a new microcentrifuge tube and centrifuged at 14000 rpm for 5 min. Aspirate 200 µL supernatant to an autosampler vial for further UPLC/Q-TOF–MS/MS analysis (Q Exactive Plus, Thermo Fisher). Chromatographic separation was carried out on an acquityuplchsst3 (2.1 mm × 100 mm × 1.8 µm) with a flow rate of 0.3 mL/min at 35℃. The mobilephase A was composed of deionized water supplement with 0.1% formic acid while mobilephase B was composed of ACN, with a gradient elution procedure (Additional file 4: Table S1). UPLC/Q-TOF–MS/MS settings were as follows: The resolution for MS1 was 70,000 and 17,500 for MS2. Ion spray voltage was set to 3200 V, capillary temperature was 320 °C and Aux gas heater temperature was 350 °C. Sheath gas flow rate was set to 40 L/min and Aux gas flow rate was set to 15 L/min. Samples were analyzed in both positive and negative ionization modes with a scanning mas-to-charge (m/z) range from 100 to 1200. Compound discover 3.2 software was utilized to analyze the acquired raw data. The mzcloud combined with mzVault database were used for the identification of the components.

### Network pharmacology analysis

PubChem database(https://pubchem.ncbi.nlm.nih.gov/) was utilized to search the main components of SJS identified by mass spectrum. Traditional Chinese Medicine Systems Pharmacology (TCMSP, https://tcmspw.com/tcmsp.php) combined with Swiss Target Prediction databases was used to search the targets associated with the active ingredients. Genecards database, with “sepsis” as the key word, were used to collect the disease-associated targets. Draw Venn Diagram was used to obtain their overlapped targets. The database for Annotation, Visualization, and Integrated Discovery (DAVID, https://david.ncifcrf.gov/) was applied to determine the KEGG pathways and Gene Ontology (GO) enrichment analysis. Cytoscape 3.8 software was performed to construct the network diagrams.

### Molecular docking

The active ingredients of SJS and the crucial disease-associated targets were selected for molecular docking. The PDB files containing a 3D structure of the proteins were downloaded from the PDB database (https://www.rcsb.org/). The SDF files containing the active components were searched from the database of PubChem. AutoDock vina software was applied for molecular docking. The docking results of the proteins with their corresponding ligands were presented with the docking scores. The 3D structure was visualized using PyMOL software.

### Cell culture

MH-S cells were cultured in RPMI 1640 medium supplemented with 10% fetal bovine serum (FBS) and 1% streptomycin /penicmin at 37 °C in an environment of 5% CO_2_ and stimulated with 5 µg/mL LPS for 4 h together with 50 µmM ATP for 2 h. CCK8 was used to detect the cell viability.

### Enzyme-linked immunosorbent assay analysis

IL-1β, IL-6, TNF-α and IL-10 levels in the sera and culture medium were examined via Enzeme-linked immunosorbent assay (ELISA), according to the manufacturer’s instructions. In our commercial ELISA kit, the monoclonal anti-IL-1β, anti-IL-6, anti-TNF-α and anti-IL-10 antibodies were pre-coated in the 96-well microplates for 24 h. Then add the samples and standards to the 96-well microplates, followed by the addition of their corresponding antibodies. Subsequently, aspirate the substrate solution to the well and measure the absorbance at the 450 nm.

### Protein preparation and western blot

Radioimmunoprecipitation assay (RIPA) was utilized to extracted and harvested proteins of the MH-S cells and BCA assay was used measure the protein concentration. Total protein (20 μg per lane) was separated on 10% sodium dodecyl sulfate–polyacrylamide gel electrophoresis (SDS-PAGE) and transferred to a polyvinylidene fluoride (PVDF) membrane. Then the PVDF membrane was soaked in 5% skim milk for 1.5 h, which was dissolved in Tris buffered saline Tween (TBST). Subsequently, the soaked PVDF membrane was incubated overnight at 4 °C with a rabbit polyclonal antibody against GAPDH(CST, Cat No: 4970S), ASC(Affinity, Cat No: DF6304), NLRP3(AB Clonal, Cat No: A5652), P65(Zhengneng Bio, Cat No: 342947), P-P65(Zhengneng Bio, Cat NO: 310013), GSDMD(Zhengneng Bio, Cat No: 342947) and Pro-caspase-1(AB Clonal, Cat NO:179515) at a dilution of 1:1000, respectively. After completion, the membrane was washed with TBST three times and incubated with a secondary anti-rabbit antibody for 1 h at room temperature. Similarly, the membrane was washed with TBST three times again. Finally, specific bands were visualized using a chemiluminescent substrate and detected using the Omega Lum C Gel Imaging System (Bio-rad). Protein band intensity was measured and quantified by ImageJ software.

### RNA isolation and qRT-PCR

Total RNA was extracted from lung tissue and MH-S cells by total RNA extraction kit (TIANGEN, Beijing, China) according to the instructions recommended by the manufacture. RNA concentration was quantified spectrophotometrically. For qRT-PCR analysis, 1 μg of total RNA was applied for cDNA synthesis using the primeScriptTM RT reagent kit (Takara, Japan, Code No. RR036A). Besides, mRNA level was quantified via quantitative PCR by using the TB Green mix (Takara, Japan, Code NO. RR420A). PCR amplification was performed over 40 cycles using the StepOne system. Amplification conditions were as follows: 95 °C for 30 s, 95 °C for 5 s, 60 °C for 34 s. The level of GAPDH level functioned as an endogenous control. Quantification of relative mRNA level was quantified using the comparative CT method, normalized to endogenous GAPDH expression. The primers used in our study were listed in Additional file 5: Table S2.

### Statistical analysis

All data were presented with mean ± s.d and statistic analyzed by GraphPad Prism 9.0 software. The Student’s unpaired *t-test* was used to determine statistical significance between the two groups. Two-way analysis of variance was applied to compare the significant difference among multiple groups. *P* < 0.05 was considered statistically significant.

## Result

### SJS treatment protects mice against CLP-induced sepsis and modulates immune homeostasis

**Fig. 1:**
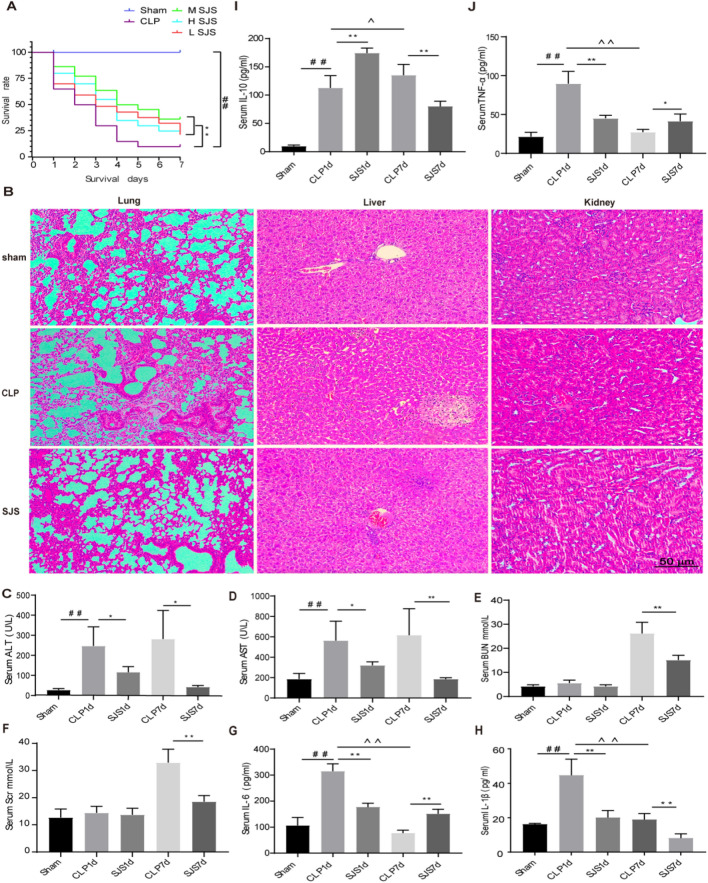
SJS treatment protects mice against CLP-induced sepsis and modulates immune homeostasis. **A**: SJS treatment improved the survival rate of sepsis mice (n = 20); **B**: SJS alleviated the multiple organ injury induced by sepsis using HE staining (n = 4); **C**-F: ALT, AST, Scr and BUN contents in SJS-treated mice using biochemical analysis (n = 4). **G**-I: Pro-inflammaory cytokines contents in SJS-treated mice using biochemical analysis (n = 4); **J**: Anti-inflammaory cytokines contents in SJS-treated mice using biochemical analysis (n = 4), *P < 0.05, **P < 0.01, ^#^*P* < 0.05, ^##^*P* < 0.01, *^P* < 0.05,^^* P* < 0.01

To explore the therapeutic effect of SJS exerted on sepsis, we subjected SJS- or NS-treated C57BL/6 mice to CLP and sham surgery and investigated its protective effects through survival rate, biochemical analysis and HE staining. Compared with the control group, significantly more C57BL/6 mice died after CLP surgery after 7 days, whereas the SJS-treated mice showed a strong decrease. Additionally, the moderate-dose group showed a better protective role than the low- or high- dose group during the whole sepsis progression (Fig. 1A). Therefore, 6.75 g/kg SJS was used in our further study.

**Fig. 2:**
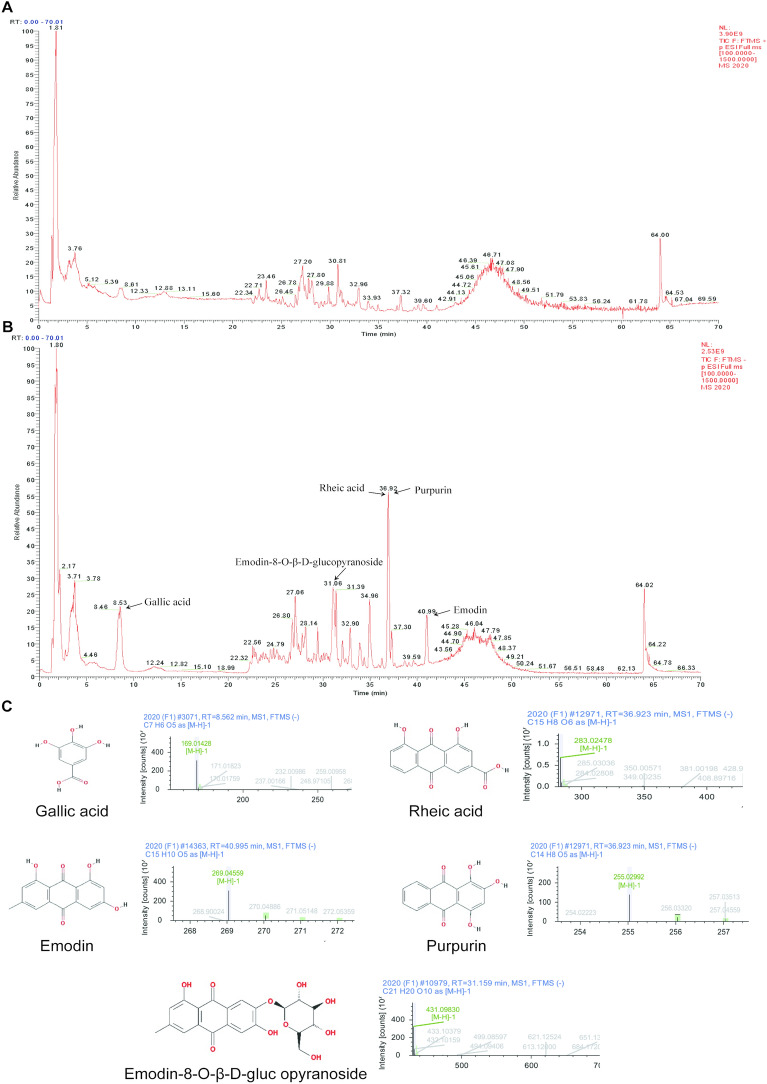
Chemical components identification using UPLC/Q-TOF–MS/MS. **A**–**B**: The total ion chromatograms (TICs) of SJS in a positive or negative ion mode; **C**:Chemical structures and secondary mass spectra of Gallic acid, Rheic acid, Emodin, Purpurin and Emodin-8-O-β-d-Gluc opyranoside

Next, we investigated the protective role moderate-dose of SJS exerted on sepsis-induced multiple organ injury. Our biochemical analysis showed that the contents of AST and ALT, rather than SCr and BUN, were significantly elevated at day 1 post CLP, compared to those of the sham group. Besides, all of these biomarkers were elevated significantly at day 7, which can be decreased by SJS treatment (Fig. 1B–E). HE staining results were highly similar to those obtained by biochemical analysis (Fig. 1F). Additionally, ELISA data from Fig. 2G–J showed that SJS could decrease the release of pro-inflammatory cytokines while increase the production of anti-inflammatory cytokines at day 1. Interestingly, when developed into the immunosuppression phase, SJS acted to modulate the cytokines release and boost immune response (Fig. 1G–J). Accumulately, our data demonstrated that SJS treatment protects mice against CLP-induced sepsis and modulates immune homeostasis.

### Chemical components identification

To make clear the potential mechanism, we firstly identified the chemical components of SJS using UPLC/Q-TOF–MS/MS analysis both in a positive and negative mode (Fig. 2A–B). A total of 96 compounds were observed, including 23 acids, 16 flavonoids, 10 anthraquinones, 11 esters, 6 sugars, 6 phenols, 5 aldehydes, 5 alcohols, 3 amino cids and 11 others (Additional file 6: Table S3). Previous studies showed demonstrated that Gallic acid, Rheic acid, Emodin, Purpurin and Emodin-8-O-β-D-glucopyranoside acted to inhibit inflammatory response and adjust immunity [18–23], which were the top 20 compounds in SJS.

### Network pharmacology analysis

**Fig. 3:**
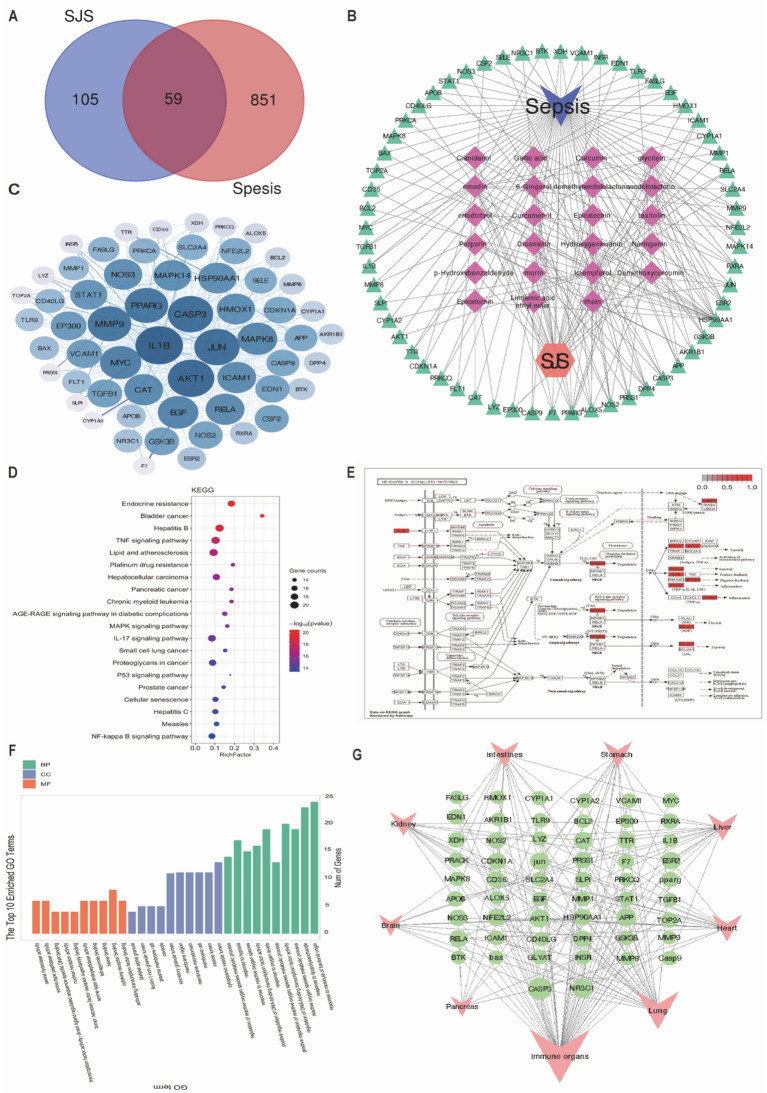
The potential molecular mechanism of SJS to treat sepsis based on network pharmacology analysis. **A**: Veen diagram between the targets of SJS and disease. **B**: Ingredients—genes—diseases network diagram; **C**: PPI network diagram; **D**: KEGG pathway enrichment; **E**: Visual analysis of NF-kappa B signaling pathway; **F**: GO enrichment analysis; **G**: Tissue expression analysis of intersection genes

Through the TCMSP databases, a total of 23 active components were collected (Additional file 7: Table S4). Subsequently, disease-associated targets were obtained from the disease-associated database and the targets of the active ingredients were screened out via the databases of TCMSP and Swiss Target Prediction. After matching the 164 SJS-associated proteins with 910 sepsis-related targets, 59 shared targets were identified as potential targets for SJS to treat sepsis (Fig. 3A).

To screen out the hub genes and crucial compounds of SJS against sepsis, we performed ingredients-genes-disease and PPI networks through Cytoscape 3.8. Figure 3B revealed the major active components of SJS,such as kaempferol, emodin, glycitein, and morin etc., suggesting that they were the crucial compounds of SJS to treat sepsis. Figure 3C uncovered the hub genes based on the degree value, including IL-1β (degree = 47), RELA (degree = 32) and so on, indicating these genes were the core targets.

To decipher the molecular mechanism of the identified potential targets, 59 overlapping targets were submitted to DAVID database for GO enrichment and KEGG pathway analysis. We totally obtained 146 pathways by KEGG enrichment analysis, and we further visualized the top 20 pathways (Fig. 3D), such as NF-kappa B signaling pathway (Fig. 3E). In addition, a total of 1158 terms in GO function enrichment analysis were acquired, including 51 terms in Molecular Function (MF), 18 in Cellular Component (CC) and 1089 in Biological Process (BP) Visual analysis revealed that MF mainly were involved in transcription factor binding, cytokines receptor binding, heme binding; CC were involved in cytoplasmic vesicle lumen, vesicle lumen, membrane raft; and BC were involved in molecule of bacterial origin, reactive oxygen species metabolic process, lipopolysaccharide (Fig. 3F) Multiple organ injury is leading cause of death induced by sepsis. Next, we imported the 59 overlapped genes into the Bio GPS database for tissue expression analysis. Our data demonstrated that the overlapped targets were mainly involved in immune organs and lung, suggesting that SJS may exert a protective role in sepsis-induced lung injury (Fig. 3G).

### Molecular docking

**Fig. 4:**
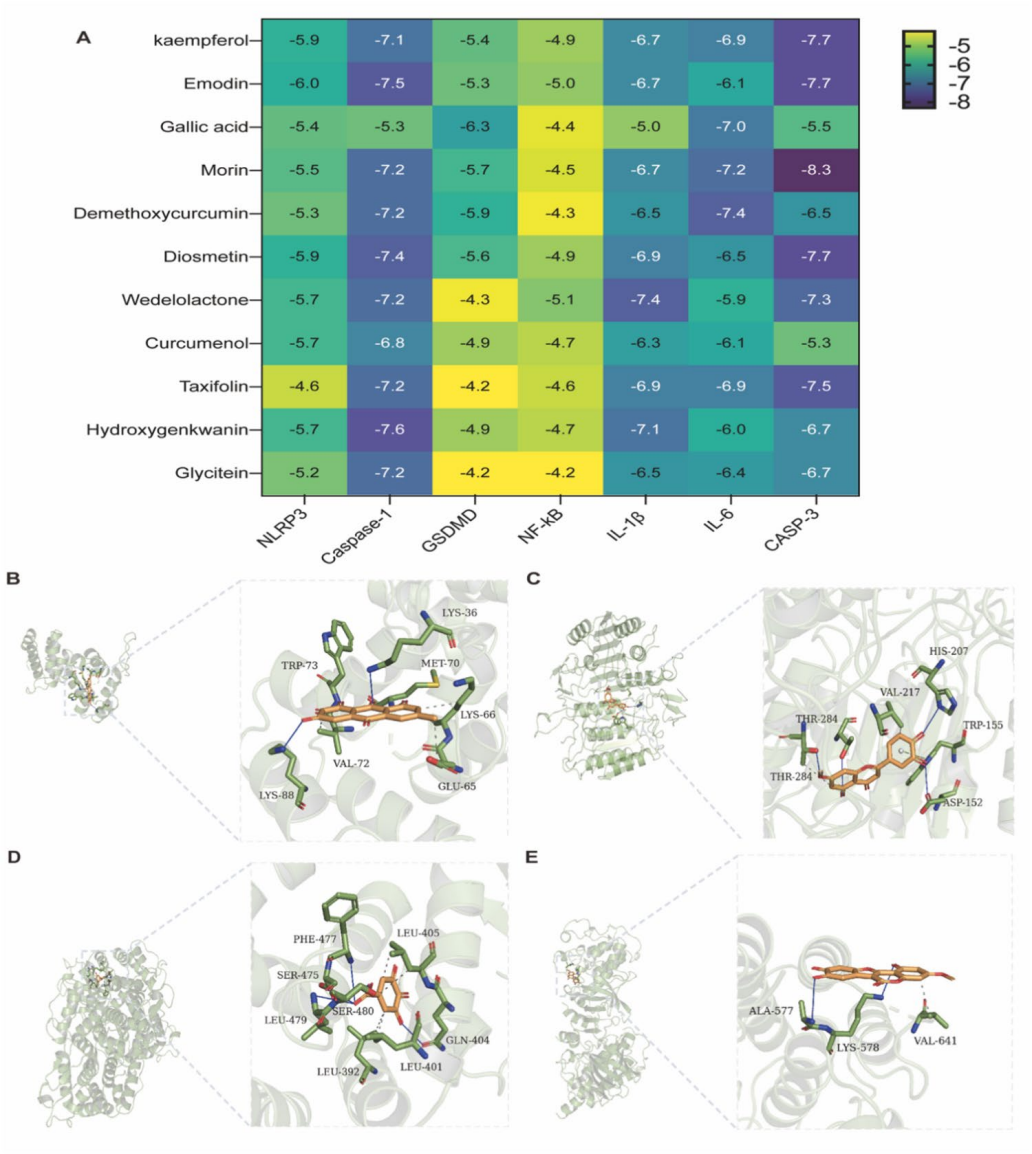
Molecular docking between the active ingredients and pyroptosis-associated proteins. **A**: Binding affinity between the major active ingredients and pyroptosis-assocaited proteins; **B**–**E**: 3D docking diagram of Emodin with NLRP3 (**B**), Hydroxygenkwanin with caspase-1 (**C**), gallic acid with GSDMD (**D**) and NF-kB with Wedelolactone (**D**), the light dashed lines represent hydrogen bonds, the dark dashed lines demarcate π–π interactions

Our bioinformatics analysis has validated that NF-kB and and IL-1β were the core genes, which also were the key components of pyroptosis. Therefore, we next wanted to explore the binding affinity between the major active ingredients and pyroptosis-assocaited proteins. The absolute binding energy value > 4.25 kcal/mol was considered a good binding activity. The heat map results of our molecular docking study implicated that the most ingredients have a strong affinity with the pyroptosis-associated proteins, such as NLRP3, Caspase-1, GSDMD and NF-kB (Fig. 4A–E). These data implicated that SJS perhaps exerted its therapeutical effect through the regulation of pyroptosis.

### SJS bidirectional modulated pyroptosis through the regulation of NF-kB/NLRP3 axis

**Fig. 5:**
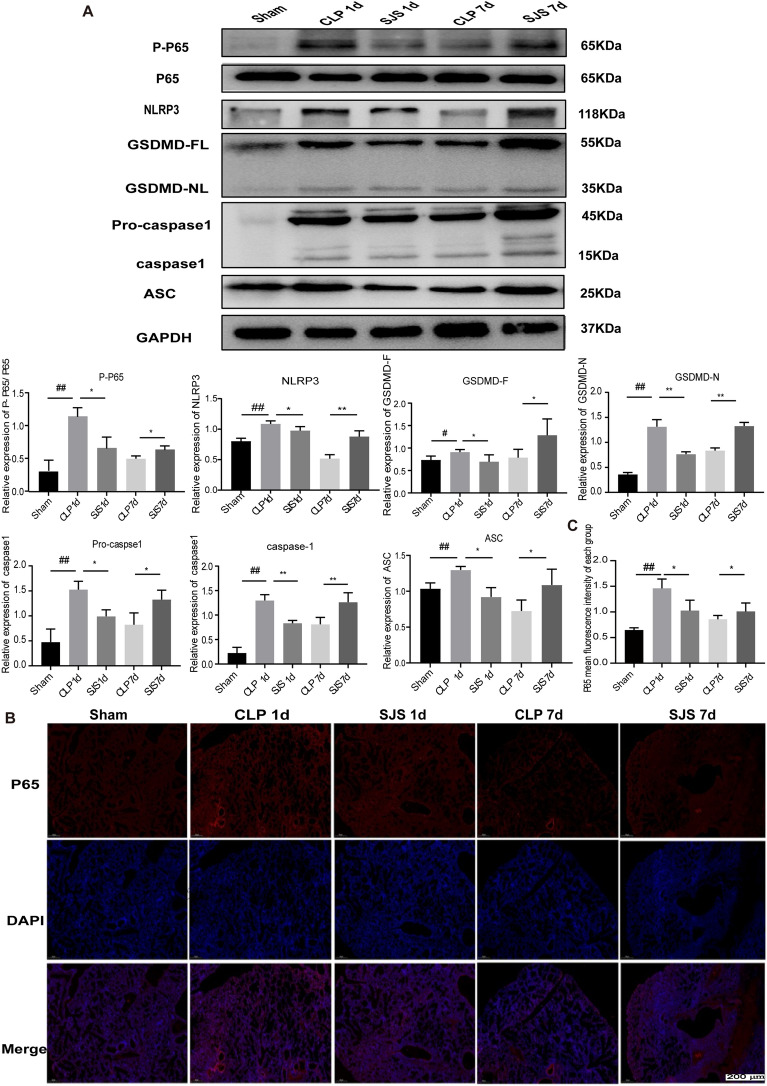
SJS bidirectional modulated pyroptosis through the regulation of NF-kB/NLRP3 axis. **A**: The protein levels of P-P65, NLRP3, GSDMD, caspase-1 and ASC using western blot (n = 4). **B**–**C**: SJS modulated the translocation of p65 to the nucleus using immunofluorescence staining (n = 3) *P < 0.05, **P < 0.01, ^#^*P* < 0.05, ^##^*P* < 0.01

Our previous study revealed that SJS functioned to maintain immune homeostasis, but its potential molecular mechanism is still largely unknown. As our molecular docking results revealed SJS presented with a good affinity for the pyroptosis-associated proteins, next, we wanted to explore whether SJS could regulate pyroptosis homeostasis. We conducted western blot, immunofluorescence staining and qRT-PCR of the sorted murine lung at different time points (0, 1 and 7d) after sepsis. These three time points represented the major stages in the dynamic alternation of sepsis progression, corresponding to a continuous inflammatory stimuli and immunosuppression. As shown in Fig. 5A, SJS was able to decrease the levels of GSDMD, caspase-1, ASC in the continuous inflammatory phase. Further detection found SJS intervention up-regulated their expression in the immunosuppression stage. Our qRT-PCR results is highly similar with the data of western blot assay (Additional file 1: Fig. S1). Together, these results indicated SJS exerted a bidirectional regulatory effects in pyroptosis.

Several studies reported that NF-kB/NLRP3 axis is an effective and functionally relevant regulator of pyroptosis. Therefore, we detected the protein levels of NLRP3 and NF-kB. Figure 5A, B demonstrated that NLRP3、P-P65 and P65 levels was increased in the early stage while decreased in the immunosuppression phase, all of which partially reversed by SJS treatment. Together, these data showed the ability of SJS to tune immune homeostasis back toward the center between the extremes of the excessive inflammatory response or immunosuppression stage through NF-kB/NLRP3/pyroptosis axis.

### SJS alleviated LPS-induced inflammation in alveolar macrophages by modulating pyroptosis through NF-kB/NLRP3 axis

**Fig. 6:**
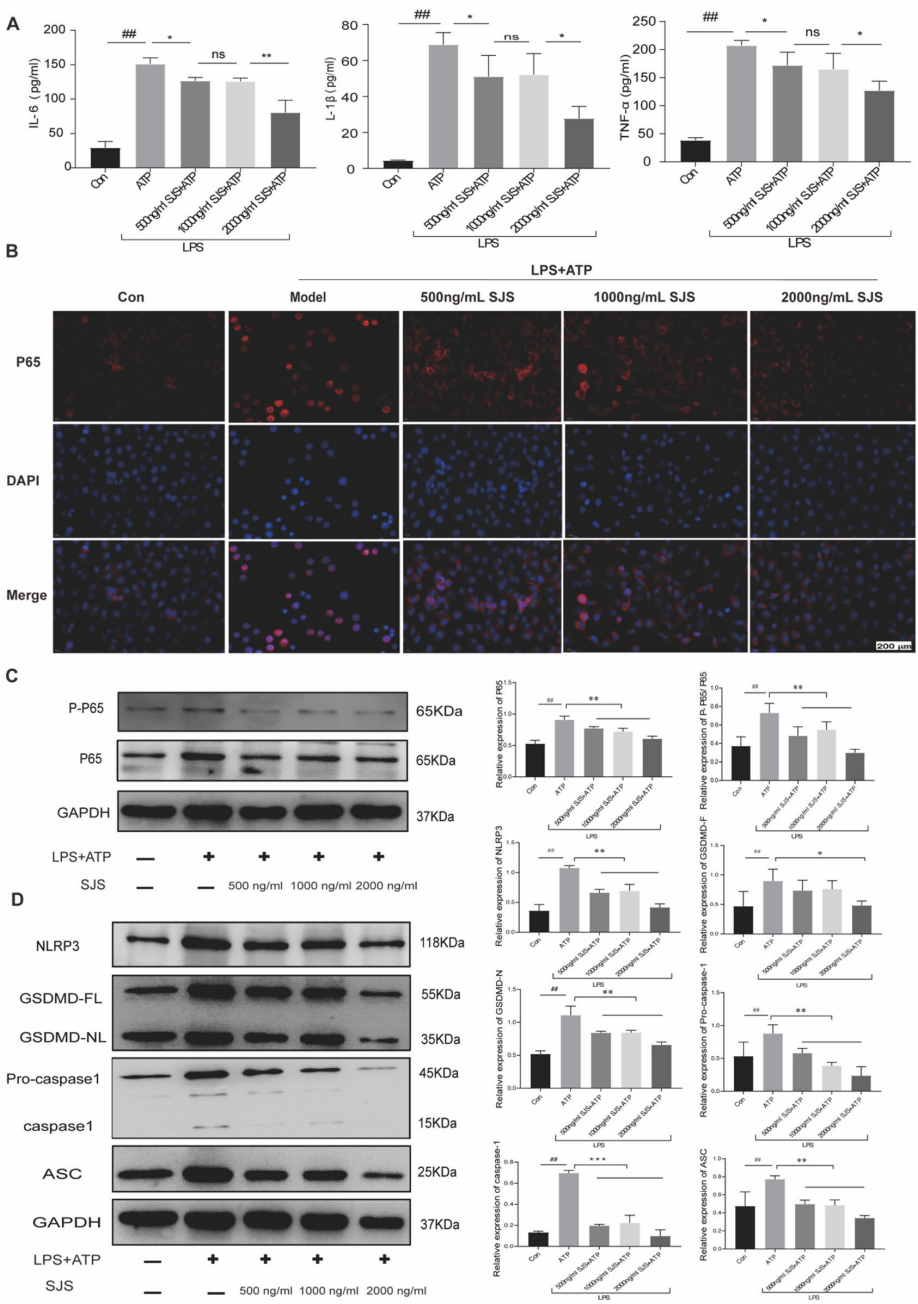
SJS alleviated LPS-induced inflammation in alveolar macrophages by modulating pyroptosis through NF-kB/NLRP3 axis. **A**: IL-6, IL-1β and TNF-α contents in culture medium using ELISA assay (n = 4); B: P65 level (n = 4); **C**–**D**: P-P65, NLRP3, GSDMD, caspase-1 and ASC protein level (n = 4). *P < 0.05, **P < 0.01, ^#^*P* < 0.05, ^##^*P* < 0.01

To validate the effects SJS exerted on pytoptosis, MH-S cells were used to evaluate its regulatory role. Firstly, we detected the cell viability of MH-S cells treated with different concentration of SJS (0, 500, 1000, 2000, 4000 ng/mL) under normal conditions using CCK8 assays. Our results revealed that 4000 ng/mL SJS could significantly decrease the cells viability (Additional file 2: Fig. S2). Therefore, 0, 500, 1000, and 2000 ng/mL of SJS were utilized in our follow-up study. Then, MH-S cells were treated with 5 µg/mL LPS together with 50 µmoL ATP followed by different concentrations of SJS. Our ELISA results uncovered the release of pro-inflammatory cytokines, such as IL-1β, TNF-α, and IL-6 were elevated significantly by LPS and ATP stimuli, which could be effectively reduced after the addition of SJS (Fig. 6A).

As we have verified that SJS is able to modulate pyroptosis in sepsis animal model, next, we detected the protein level of GSDMD, and caspase-1 of MH-S cells treated with LPS and ATP along with different concentrations of SJS. Our in vitro results demonstrated the expression levels of these proteins were dramatically increased under LPS and ATP stimuli, which can be reversed by different concentrations of SJS (Fig. 6D). The results of qRT-PCR are shown in Additional file 3: Fig. S3. Together, these findings suggested that SJS could modulate pyroptosis of alveolar macrophages at cellular level.

To elucidate the mechanism involved in lung injury in vitro, we cultured MH-S cells and detected the expression level of NF-kB/NLRP3 axis, a crucial regulator of pyroptosis. As shown in Fig. 6B–D, P-P65、P65 and NLRP3 expression levels were significantly increased under LPS and ATP stimuli, which was attenuated in SJS-treated MH-S cells. These data suggested SJS inhibited pyroptosis of MH-S cells through NF-kB/NLRP3 axis.

**Fig. 7:**
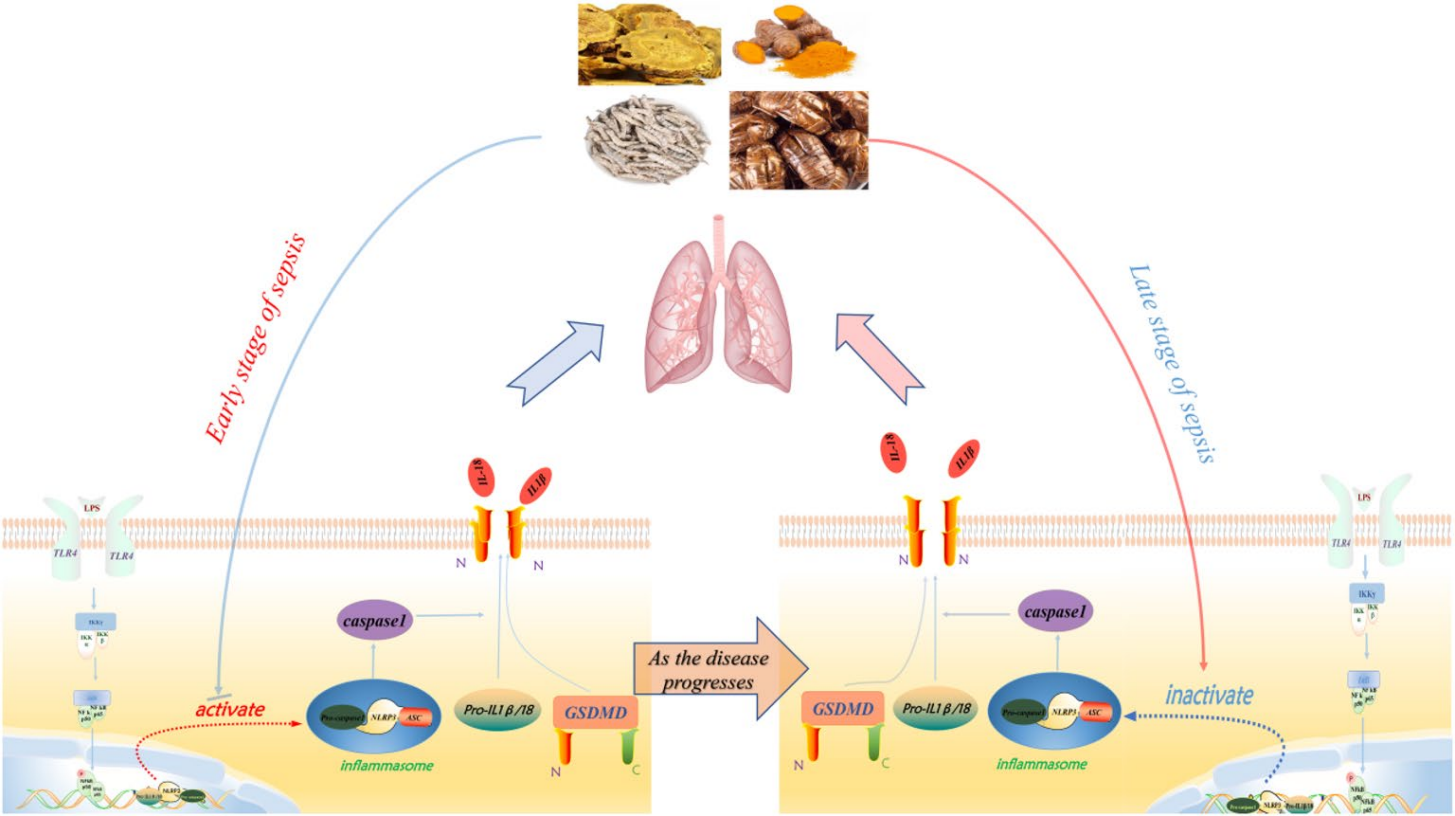
Shengjiang San alleviated sepsis-induced lung injury through its bidirectional regulatory effect. In the continuous inflammatory phase, SJS could inhibit the NF-kB/NLRP3 axis and suppress the pyroptosis, thus alleviating inflammatory response. As the disease progresses, immunosuppression was seen and SJS could enhance the NF-kB/NLRP3 axis and promote the pyroptosis, thus boosting immune response

## Discussion

Sepsis is a life-threatening complex disease caused by the host's dysfunctional response to infection [24]. The pathological process of this disease can be divided into two stages [25]. In the early stage, the immune system is activated by various pathogenic microorganisms, which causes the over-production of pro-inflammatory factors, thus leading to the occurrence of cytokines storm and multiple organ dysfunction [26, 27]. In the late stage, due to the dysfunction of antigen presenting cells and increased apoptosis of lymphocytes, pro-inflammatory factors production decreased while anti-inflammatory cytokines release increased, immunosuppression was observed [28, 29]. Both cytokines storm and immunosuppression were reported to be the main cause of low survival rate [30]. Nowadays, therapeutic strategies target for sepsis either focused on the uncontrolled inflammatory response stage or the immunosuppression phase, ant its therapeutic efficacy is still not satisfactory [31]. Therefore, it is urgent for us to find several novel drugs possessed bidirectional regulation function. As a well-known traditional Chinese medicine, SJS acted to maintain immune homeostasis, but its underlying therapeutic mechanism in sepsis is still unclear. In this study, SJS can improve the 7-day survival rate of sepsis mice, inhibit the release of inflammatory cytokines IL-1β, IL-6, TNF-α, and increase the production of IL-10 in the early stage. Furthermore,when develops into the immunosuppression phase, SJS could increase these pro-inflammatory factors release and inhibit IL-10 production, indicating it functioned to modulate immune balance. In addition, we also found SJS could protect mice against CLP-induced multiple organ injury. These results provide a basis for us to further explore its underlying therapeutic mechanism in the treatment of sepsis.

As a classical formula, SJS is consisted by several herbs while its specific active components are still unclear. In the present work, we firstly identified the major compounds of SJS and totally obtained 96 ingredients using UPLC/Q-TOF–MS/MS approaches, most of which acted to inhibit inflammation and modulate immune response,such as curcumin and emodin. Curcumin is reported to participate in various immune reactions through regulating the activation of immune cells and the production of cytokines [32]. Experimental studies demonstrated emodin was an effective therapeutic drug in the treatment of sepsis for its role in alleviating the damage of intestinal barrier, restoring the microecological balance of intestinal flora, promoting intestinal peristalsis, as well as repressing the expression of IL-6, IL-8, TNF-α in lung tissue [33, 34]. These data implicated the components diversity of SJS perhaps exerted protective role in the whole sepsis progression through different mechanisms. Our network pharmacology analysis revealed SJS treated sepsis through multiple pathways and multiple targets, including NF-kB pathway, TNF signaling pathway and IL-17 signaling pathway, which is reported to be closely related to the inflammation and immunity. PPI network diagram showed that the core targets were IL-1β, RELA, etc. Among the hospitalized patients, ALI is closely correlated with higher mortality of sepsis. Our further bioinformatics analysis revealed these core genes were highly expressed in lung tissues and closely related to NF-kB signaling pathways. As IL-1β and NF-kB were the crucial components of pyroptosis [35], we speculated that SJS may exerted protective role in sepsis-induced lung injury through the regulation of pyroptosis. Additionally, our molecular docking results validated the active ingredients of SJS have a strong affinity for the pyroptosis-associated proteins.

Pyroptosis is a new form of programmed cell death characterized by swelling of cells, loss of plasma membrane integrity and release of inflammatory mediators, including IL-1β, TNF and IL-6. Our results showed SJS could decrease the release of these indicators in the early stage while promote their production in the late phase. A key feature of pyroptosis is caspase-mediated cleavage of a protein called gasdermin D, which leads to formation of membrane pores. Pyroptosis is majorly induced by activation of the canonical inflammasomes that use caspase-1. Our western blot assays demonstrated SJS could inhibit GSDMD and caspase-1 protein level at day 1 post CLP, while promote their expression levels at day 7. These data suggested that SJS is able to bidirectional regulated pyroptosis in sepsis progression.

NF-kB/NLRP3 axis was the crucial regulator of pyroptosis. Inhibition of NF-kB is reported to be an effective target for alleviating sepsis-induced muptiple organ dysfunction [36]. Previous studies have reported that NF-kB is a key transcriptional factor of pro-caspase-1, which functioned to enhance its transcription. Our data observed a strong increase in the expression level of pro-caspase-1 [37]. NF-kB/NLRP3 axis could promote the cleavage of pro-caspase-1, thus leading to the up-regulation of caspase-1, GSDMD and the increased secretion of IL-1β, thus resulting in a strong increase in pyroptosis [38]. Therefore, modulating NF-kB/NLRP3 axis may be a key strategy for the treatment of sepsis (Fig. 7).

As imbalanced immune response in lung usually induced by macrophages, in our study, MH-S macrophage cell line was selected for the further study. As LPS and ATP are essential for pyroptosis and the roles they exerted are different. LPS is reported to be involved in the priming process while ATP plays an crucial role in the activation process [38]. Therefore, in our following study, we used LPS together with ATP to establish a cells model. Our results revealed the cell viability was sharply decreased when treated with 4000 ng/mL of SJS, whereas no significantly difference was observed in 0, 500,1000, or 2000 ng/mLSJS treated group. These data suggested high-dose of SJS could lead to some side effects. This phenomenon was also observed in mice as the survival rate of sepsis mice treated with high-dose of SJS did not increase, but decrease instead, compared to the moderate-dose group. Therefore, in our followed study, 05001000,and 2000 ng/mL of SJS were used. Our results showed SJS could modulate NLRP3, P-P65, GSDMD, and Caspase-1 protein levels in vitro. These data verified that SJS is able to regulate pyroptosis and pyroptosis-assocaited proteins at cellular level.

In conclusion, we observed the therapeutic effects of SJS during the whole sepsis progression and found that SJS could tune immune homeostasis back toward the center between the extremes of the excessive inflammatory response and immunosuppression stage through the regulation of pyroptosis.

## Supplementary Information

**Additional file 1: Fig. S1**. SJS bidirectionally regulates immune homeostasis in lung injury with sepsis. A-F The mRNA levels of NLRP3, caspase-1, IL-1β, IL-6, IL-10, PD-1 using qRT-PCR (n=4) *P<0.05, **P<0.001, ns no significant difference.

**Additional file 2: Fig. S2**. The cell viability of MH-S cells treated with different concentration of SJS. ^#^*P<0.05*.

**Additional file 3: Fig. S3**. SJS alleviated LPS-induced inflammation in alveolar macrophages by modulating pyroptosis. A-C The mRNA levels NLRP3, caspase-1, IL-1β using qRT-PCR (n=4) **P<0.01, ^##^*P<0.01.*

**Additional file 4: Table S1**. Gradient elution condition.

**Additional file 5: Table S2.**The specific RT primers were listed as below.

**Additional file 6: Table S3.** Identification of 96 chemical components from SJS by UPLC–Q/TOF–MS.

**Additional file 7: Table S4.** 23 Active Compounds.

## Data Availability

Data sets used and/or analyzed during the current study period are available from corresponding authors upon reasonable request.

## Abbreviations

SJS: Shengjiang San
CLP: Cecal ligation and puncture
GSDMD: Gasdermin D
NLRP3: NOD-like receptor thermal protein domain associated protein 3
ASC: Apoptosis-associated speck-like protein containing a CARD
Pro-caspase-1: Pro-cysteinyl aspartate specific proteinase-1
caspase-1: Cysteinyl aspartate specific proteinase-1
IL-6: Interleukin-6
IL-10: Interleukin-10
IL-1β: Interleukin-1β
TNF-a: Tumor necrosis factor alpha
HE: Hematoxylin–eosin
AST: Aspartate aminotransferase
ALT: Alanine aminotransferase
BUN: Blood urea nitrogen
Scr: Serum creatinine
LPS: Lipopolysaccharide
ATP: Adenosine-triphosphate

## References

[1] Genga KR, Russell JA (2017). Update of Sepsis in the intensive care unit. J Innate Immun.

[2] Venet F, Monneret G (2018). Advances in the understanding and treatment of sepsis-induced immunosuppression. Nat Rev Nephrol.

[3] Liu D, Huang SY, Sun JH, Zhang HC, Cai QL, Gao C, Li L, Cao J, Xu F, Zhou Y (2022). Sepsis-induced immunosuppression: mechanisms, diagnosis and current treatment options. Mil Med Res.

[4] Cao C, Yu M, Chai Y (2019). Pathological alteration and therapeutic implications of sepsis-induced immune cell apoptosis. Cell Death Dis.

[5] Coll RC, Schroder K, Pelegrín P (2022). NLRP3 and pyroptosis blockers for treating inflammatory diseases. Trends Pharmacol Sci.

[6] Wu M, Yang Z, Zhang C, Shi Y, Han W, Song S, Mu L, Du C, Shi Y (2021). Inhibition of NLRP3 inflammasome ameliorates podocyte damage by suppressing lipid accumulation in diabetic nephropathy. Metabolism.

[7] Mangan MSJ, Olhava EJ, Roush WR, Seidel HM, Glick GD, Latz E (2018). Targeting the NLRP3 inflammasome in inflammatory diseases. Nat Rev Drug Discov.

[8] Huang Y, Xu W, Zhou R (2021). NLRP3 inflammasome activation and cell death. Cell Mol Immunol.

[9] Zhao N, Li CC, Di B, Xu LL (2020). Recent advances in the NEK7-licensed NLRP3 inflammasome activation: Mechanisms, role in diseases and related inhibitors. J Autoimmun.

[10] Kesavardhana S, Malireddi RKS, Kanneganti TD (2020). Caspases in cell death, inflammation, and pyroptosis. Annu Rev Immunol.

[11] Abhimanyu O, Guerra-Resendez CO, Nishiguchi RS, Ladki T, Hilton M, Schlesinger IB, DiNardo LS (2021). Reversing post-infectious epigenetic-mediated immune. Suppression Front Immunol.

[12] Zhou G (2012). Zhang Hc, Gong X: Clinical study on the treatment of systemic inflammatory response syndrome in patients with severe disease. Chin Tradit Med Emerg.

[13] Zhu Y, Ouyang Z, Du H, Wang M, Wang J, Sun H, Kong L, Xu Q, Ma H, Sun Y (2022). New opportunities and challenges of natural products research: When target identification meets single-cell multiomics. Acta Pharm Sin B.

[14] Fan TT, Cheng BL, Fang XM, Chen YC, Su F (2020). Application of Chinese medicine in the management of critical conditions: a review on sepsis. Am J Chin Med.

[15] Zhu L, Xi Y, Zhao L (2017). The intervention effect of Lifingsan on Th17/Treg imbalance and related regulatory factors in patients with sepsis. J Clin Emerg.

[16] van Gelder T, Klupp J, Barten MJ, Christians U, Morris RE (2001). Comparison of the effects of tacrolimus and cyclosporine on the pharmacokinetics of mycophenolic acid. Ther Drug Monit.

[17] Rittirsch D, Huber-Lang MS, Flierl MA, Ward PA (2009). Immunodesign of experimental sepsis by cecal ligation and puncture. Nat Protoc.

[18] Dengke L, Qingwen M, Zhenxiao S (2015). Research progress in pharmacological action of emodin-8-O-β-D-glucopyranoside. Chin J Pharmacol Toxicol.

[19] Al Zahrani NA, El-Shishtawy RM, Asiri AM (2020). Recent developments of gallic acid derivatives and their hybrids in medicinal chemistry: a review. Eur J Med Chem.

[20] Dong X, Fu J, Yin X, Cao S, Li X, Lin L, Ni J (2016). Emodin: a review of its pharmacology, toxicity and pharmacokinetics. Phytother Res.

[21] Cao Q, Guo Y, Ye L (2012). Research progress on the anti-inflammatory effect and mechanism of Rhubarb and its active ingredients. Chin Herbal Med.

[22] Singh J, Hussain Y, Luqman S, Meena A (2020). Purpurin: A natural anthraquinone with multifaceted pharmacological activities. Phytother Res..

[23] Yu M, Chen TT, Zhang T, Jia HM, Li JJ, Zhang HW, Zou ZM (2021). Anti-inflammatory constituents in the root and rhizome of Polygonum cuspidatum by UPLC-PDA-QTOF/MS and lipopolysaccharide-activated RAW2647 macrophages. J Pharm Biomed Anal.

[24] Pierrakos C, Velissaris D, Bisdorff M, Marshall JC, Vincent JL (2020). Biomarkers of sepsis: time for a reappraisal. Crit Care.

[25] McBride MA, Patil TK, Bohannon JK, Hernandez A, Sherwood ER, Patil NK (2020). Immune checkpoints: novel therapeutic targets to attenuate sepsis-induced immunosuppression. Front Immunol.

[26] Grondman I, Pirvu A, Riza A, Ioana M, Netea MG (2020). Biomarkers of inflammation and the etiology of sepsis. Biochem Soc Trans.

[27] Lelubre C, Vincent JL (2018). Mechanisms and treatment of organ failure in sepsis. Nat Rev Nephrol.

[28] Zhirnov OP (2020). Molecular Targets in the Chemotherapy of Coronavirus Infection. Biochemistry.

[29] Nolt B, Tu F, Wang X, Ha T, Winter R, Williams DL, Li C (2018). Lactate and Immunosuppression in Sepsis. Shock.

[30] Denstaedt SJ, Singer BH, Standiford TJ (2018). Sepsis and Nosocomial Infection: Patient Characteristics, Mechanisms, and Modulation. Front Immunol.

[31] Gotts JE, Matthay MA (2016). Sepsis: pathophysiology and clinical management. BMJ.

[32] Rahiman N, Markina YV, Kesharwani P, Johnston TP, Sahebkar A (2022). Curcumin-based nanotechnology approaches and therapeutics in restoration of autoimmune diseases. J Control Release.

[33] Shang L, Liu Y, Li J, Pan G, Zhou F, Yang S (2021). Emodin protects sepsis associated damage to the intestinal mucosal barrier through the VDR/ Nrf2 /HO-1 pathway. Front Pharmacol.

[34] Li X, Shan C, Wu Z, Yu H, Yang A, Tan B (2020). Emodin alleviated pulmonary inflammation in rats with LPS-induced acute lung injury through inhibiting the mTOR/HIF-1α/VEGF signaling pathway. Inflamm Res.

[35] Busch K, Kny M, Huang N, Klassert TE, Stock M, Hahn A, Graeger S, Todiras M, Schmidt S, Chamling B (2021). Inhibition of the NLRP3/IL-1β axis protects against sepsis-induced cardiomyopathy. J Cachexia Sarcopenia Muscle.

[36] Xu H, Ye X, Steinberg H, Liu SF (2010). Selective blockade of endothelial NF-kappaB pathway differentially affects systemic inflammation and multiple organ dysfunction and injury in septic mice. J Pathol.

[37] Staal J, Bekaert T, Beyaert R (2011). Regulation of NF-κB signaling by caspases and MALT1 paracaspase. Cell Res.

[38] Yu P, Zhang X, Liu N, Tang L, Peng C, Chen X (2021). Pyroptosis: mechanisms and diseases. Signal Transduct Target Ther.

