# Correction: Shengjiang San alleviated sepsis-induced lung injury through its bidirectional regulatory effect

**DOI:** 10.1186/s13020-023-00760-6

**Published:** 2023-05-15

**Authors:** Shifan Yan, Yu Jiang, Ting Yu, Changmiao Hou, Wen Xiao, Jing Xu, Huili Wen, Jingjing Wang, Shutong Li, Fang Chen, Shentang Li, Xiehong Liu, Hao Tan, Lianhong Zou, Yanjuan Liu, Yimin Zhu

**Affiliations:** 1 Department of Emergency, Institute of Emergency Medicine, Hunan Provincial People’s Hospital (The FirstAfliated Hospital of Hunan Normal University), 69 Jiefang Western Road, Changsha, 410000 Hunan People’s Republic of China; 2 Hunan Provincial Key Laboratory of Emergency and Critical Care Metabonomics, Changsha, Hunan China; 3 Hunan University of Chinese Medicine, Changsha, Hunan China; 4 Department of Pediatrics, Third Xiangya Hospital of Central South University, Changsha, Hunan China; 5 Jiangxi University of Chinese Medicine, Nanchang, Jiangxi China

**Correction: Chinese Medicine (2023) 18:39** 10.1186/s13020-023-00744-6

Following publication of the original article [1], the authors reported an error in the author names of Xiehong Liu and Hao Tan.

The correct author names have been updated in this correction.

The original article [1] has been corrected.
