# Supplementary figures and images for: Shengjiang San alleviated sepsis-induced lung injury through its bidirectional regulatory effect
Source: Chin Med. 2023 Apr 17;18:39. doi: 10.1186/s13020-023-00744-6 (PMC10108513; doi:10.1186/s13020-023-00744-6)

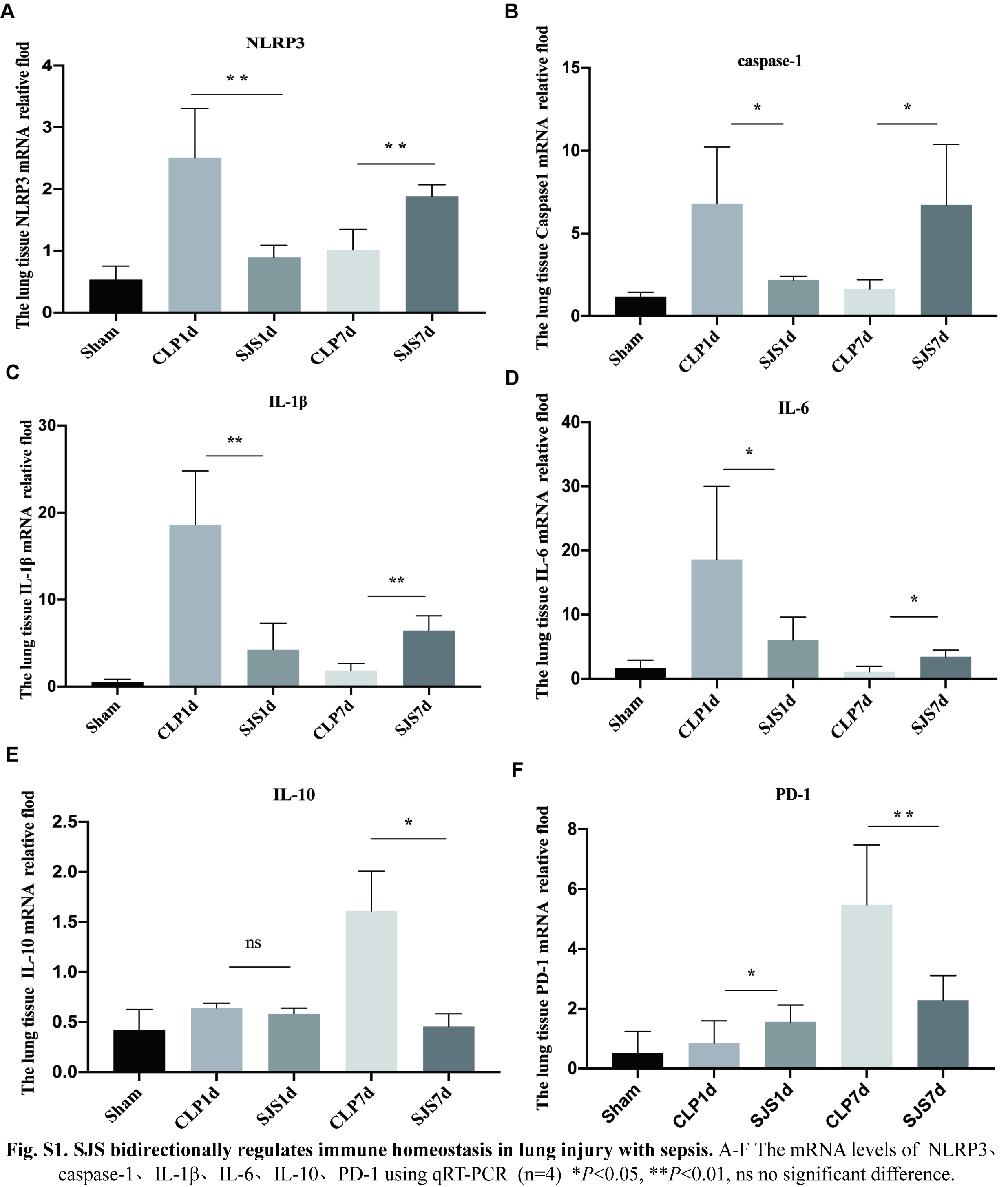

Supplement: Supplementary file 1 — Additional file 1: Fig. S1. SJS bidirectionally regulates immune homeostasis in lung injury with sepsis. A-F The mRNA levels of NLRP3, caspase-1, IL-1β, IL-6, IL-10, PD-1 using qRT-PCR (n=4) *P<0.05, **P<0.001, ns no significant difference. [file 13020_2023_744_MOESM1_ESM.tif]

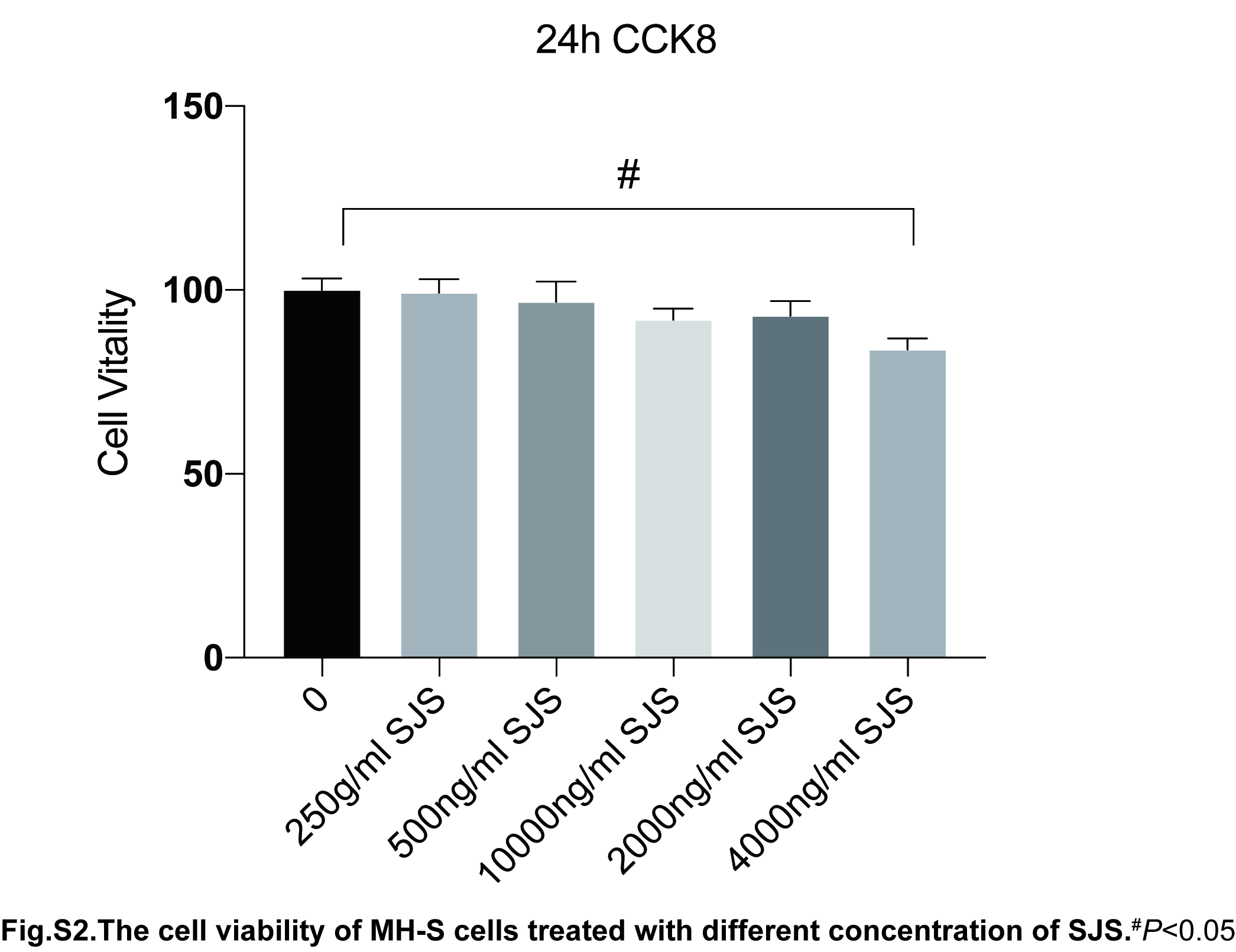

Supplement: Supplementary file 2 — Additional file 2: Fig. S2. The cell viability of MH-S cells treated with different concentration of SJS. #P<0.05. [file 13020_2023_744_MOESM2_ESM.tif]

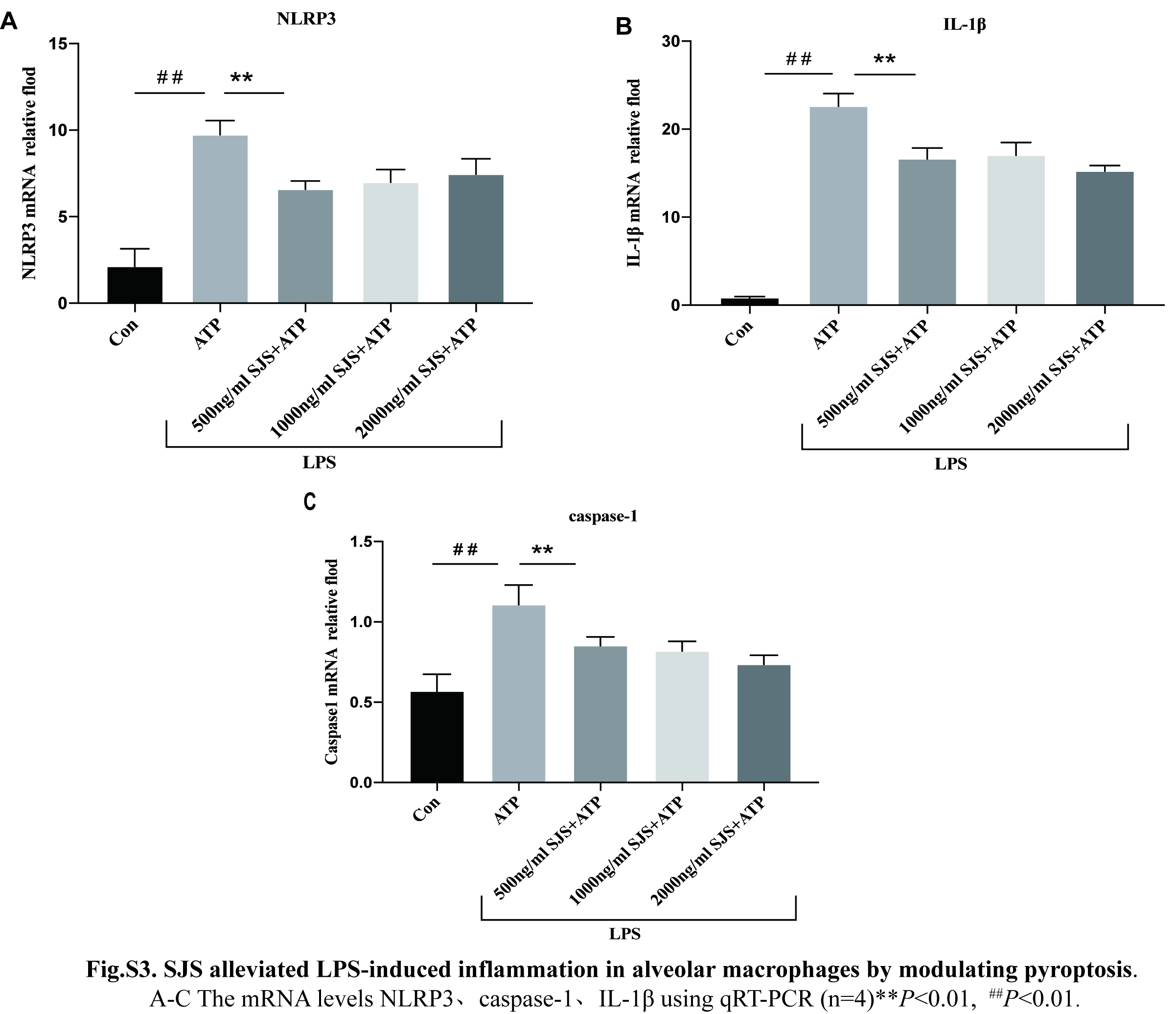

Supplement: Supplementary file 3 — Additional file 3: Fig. S3. SJS alleviated LPS-induced inflammation in alveolar macrophages by modulating pyroptosis. A-C The mRNA levels NLRP3, caspase-1, IL-1β using qRT-PCR (n=4) **P<0.01, ##P<0.01. [file 13020_2023_744_MOESM3_ESM.tif]
