# Supplementary material for: Shengjiang San alleviated sepsis-induced lung injury through its bidirectional regulatory effect
Source: Chin Med. 2023 Apr 17;18:39. doi: 10.1186/s13020-023-00744-6 (PMC10108513; doi:10.1186/s13020-023-00744-6)
Supplement: Supplementary file 4 — Additional file 4: Table S1. Gradient elution condition. [file 13020_2023_744_MOESM4_ESM.docx]

**Supplementary table 1 Gradient elution condition**

| Time | Flow Rate (mL/min) | %A | %B |
| --- | --- | --- | --- |
| 0 | 0.3 | 100 | 0 |
| 10 | 0.3 | 70 | 30 |
| 25 | 0.3 | 60 | 40 |
| 30 | 0.3 | 50 | 50 |
| 40 | 0.3 | 30 | 70 |
| 45 | 0.3 | 0 | 100 |
| 60 | 0.3 | 0 | 100 |
| 60.5 | 0.3 | 100 | 0 |
| 70 | 0.3 | 100 | 0 |
