# Supplementary material for: Shengjiang San alleviated sepsis-induced lung injury through its bidirectional regulatory effect
Source: Chin Med. 2023 Apr 17;18:39. doi: 10.1186/s13020-023-00744-6 (PMC10108513; doi:10.1186/s13020-023-00744-6)
Supplement: Supplementary file 5 — Additional file 5: Table S2.The specific RT primers were listed as below. [file 13020_2023_744_MOESM5_ESM.docx]

**Supplementary Table 2.The specific RT primers were listed as below**

| **Name of gene** | **Name of primer** | **Sequence of primers** |
| --- | --- | --- |
| NLRP3 | NLRP3-F | GGTGACCTTGTGTGTGCTTG |
|  | NLRP3-R | ATGTCCTGAGCCATGGAAGC |
| Caspase-1 | Caspase-1-F | TTATCAGGGTTGACCCCTTGG |
|  | Caspase-1-R | TTGCCCTCAGGATCTTGTCAG |
| IL-1β | IL-1β-F | GGTTCAAGGCATAACAGGCTC |
|  | IL-1β-R | TCTGGACAGCCCAAGTCAAG |
| IL-10 | IL-10-F | TGCTCTAGTCATGCTTCTG |
|  | IL-10-R | GGTTCTAAGTCGTCATTGGA |
| PD-1 | PD-1-F | TGCTGTCACTTGCTACGG |
|  | PD-1-R | ATCTTCCTTTTCCCAGTACACC |
| IL-6 | IL-6-F | AACGATGATGCACTTGCAGA |
|  | IL-6-R | GAGCATTGGAAATTGGGGTA |
| GAPDH | GAPDH-F | ACACGAGTCCTGGTGACTTTG |
|  | GAPDH-R | GGGCTTAGGTCCACACAGAA |
