# Supplementary material for: Shengjiang San alleviated sepsis-induced lung injury through its bidirectional regulatory effect
Source: Chin Med. 2023 Apr 17;18:39. doi: 10.1186/s13020-023-00744-6 (PMC10108513; doi:10.1186/s13020-023-00744-6)
Supplement: Supplementary file 6 — Additional file 6: Table S3. Identification of 96 chemical components from SJS by UPLC–Q/TOF–MS. [file 13020_2023_744_MOESM6_ESM.docx]

**Supplementary table 3 Identification of 96 chemical components from SJS by UPLC–Q/TOF–MS.**

| **Name** | **Molecular formula** | **Molecular weight** | **time** | **Peak area** | **Ion mode** |
| --- | --- | --- | --- | --- | --- |
| Citric acid | C6 H8 O7 | 192.0271 | 3.7 | 10686358077 | [M-H]^-^ |
| 2-Pyrrolidinecarboxyl ic acid | C5 H9 N O2 | 115.0635 | 1.8 | 10429949291 | [M+H]^+^ |
| Gallic acid | C7 H6 O5 | 170.0216 | 8.5 | 9761570329 | [M-H]^-^ |
| Rheic acid | C15 H8 O6 | 284.0321 | 36.9 | 9208868818 | [M-H]^-^ |
| Gluconic acid | C6 H12 O7 | 196.0583 | 1.7 | 8092678127 | [M-H]^-^ |
| Betaine | C5 H11 N O2 | 117.0791 | 1.7 | 5758706043 | [M+H]^+^ |
| Emodin-8-O-β-D-gluc opyranoside | C21 H20 O10 | 432.1056 | 31.1 | 4989280741 | [M-H]^-^ |
| Sucrose | C12 H22 O11 | 342.1163 | 1.8 | 4641845935 | [M-H]^-^ |
| DL-Arginine | C6 H14 N4 O2 | 174.1119 | 1.6 | 4262940664 | [M+H]^+^ |
| Emodin | C15 H10 O5 | 270.0529 | 41 | 4175403251 | [M-H]^-^ |
| Choline | C5 H13 N O | 103.0998 | 1.7 | 3267525700 | [M-H]^-^ |
| L-Leucine | C6 H13 N O2 | 131.0949 | 5.1 | 2764807958 | [M-H]^-^ |
| L-Pyroglutamic acid | C5 H7 N O3 | 129.0428 | 3.9 | 2735844520 | [M-H]^-^ |
| Pyrogallol | C6 H6 O3 | 126.0317 | 8.6 | 2179466474 | [M+H]+ |
| Purpurin | C14 H8 O5 | 256.0372 | 36.9 | 1813893412 | [M-H]^-^ |
| L-(-)-Malic acid | C4 H6 O5 | 134.0215 | 1.9 | 1775336591 | [M-H]^-^ |
| Uric acid | C5 H4 N4 O3 | 168.0285 | 4.1 | 1717330574 | [M-H]^-^ |
| Rubiadin | C15 H10 O4 | 254.0579 | 31.4 | 1601192899 | [M+H]^+^ |
| Chrysophanol 8-O-β-D-glucoside | C21 H20 O9 | 416.1109 | 31.4 | 1449331349 | [M-H]^-^ |
| Curcumenol | C15 H22 O2 | 234.1622 | 33 | 1037417809 | [M+H]^+^ |
| Emodin-3-methyl ether/Physcion | C16 H12 O5 | 284.0686 | 32.9 | 980521907.4 | [M-H]^-^ |
| L-Glutamic acid | C5 H9 N O4 | 147.0536 | 1.7 | 715286694.7 | [M+H]^+^ |
| Cianidanol | C15 H14 O6 | 290.0793 | 24.4 | 639604649.2 | [M-H]^-^ |
| p-Coumaric acid | C9 H8 O3 | 164.0474 | 26.8 | 636568363.8 | [M-H]^-^ |
| Demethylwedelolacto ne | C15 H8 O7 | 300.0271 | 32 | 629674460.1 | [M-H]^-^ |
| Curcumin | C21 H20 O6 | 368.1264 | 39.6 | 593967497.1 | [M+H]^+^ |
| Nicotinic acid | C6 H5 N O2 | 123.0323 | 3.1 | 556706219.7 | [M+H]^+^ |
| Mannitol | C6 H14 O6 | 182.0792 | 1.7 | 540635814 | [M-H]^-^ |
| Tinnevellin glucoside | C20 H24 O9 | 408.1422 | 30.9 | 451671393.2 | [M-H]^-^ |
| Aloeemodin | C15 H10 O5 | 270.0529 | 36.3 | 443454080.1 | [M-H]^-^ |
| Decursinol | C14 H14 O4 | 246.0894 | 30.9 | 325070062.1 | [M+H]^+^ |
| Bisdemethoxycurcum in | C19 H16 O4 | 308.1051 | 38.7 | 316832975.9 | [M+H]^+^ |
| Artemisinic acid | C15 H22 O2 | 234.1622 | 30.8 | 307684689.6 | [M+H]^+^ |
| Nicotinamide | C6 H6 N2 O | 122.0481 | 3.6 | 304549877.1 | [M+H]^+^ |
| Adenine | C5 H5 N5 | 135.0548 | 1.9 | 288893778.9 | [M+H]^+^ |
| Raffinose | C18 H32 O16 | 550.1748 | 1.8 | 283307549.9 | [M-H]- |
| Demethoxycurcumin | C20 H18 O5 | 338.1158 | 39.1 | 246366598 | [M+H]^+^ |
| Ferulic acid | C10 H10 O4 | 194.0581 | 27.5 | 239368515.7 | [M+H]^+^ |
| Epicatechin | C15 H14 O6 | 290.0793 | 25.2 | 236373676.2 | [M-H]- |
| p-Hydroxybenzaldehy de | C7 H6 O2 | 122.0368 | 25.4 | 211987697.3 | [M-H]- |
| Trigonelline HCl | C7 H7 N O2 | 137.0479 | 1.8 | 204918015.1 | [M+H]^+^ |
| Cynaroside | C21 H20 O11 | 448.1009 | 27.9 | 199353710.5 | [M-H]^-^ |
| (-)-Epicatechin gallate (-) | C22 H18 O10 | 442.0901 | 27.2 | 189780733.1 | [M-H]^-^ |
| Manninotriose | C18 H32 O16 | 504.1693 | 2.7 | 187246820.9 | [M-H]^-^ |
| Protocatechualdehyde | C7 H6 O3 | 138.0318 | 23.7 | 178810894.4 | [M-H]^-^ |
| Quinic acid | C7 H12 O6 | 192.0634 | 1.8 | 176024614.9 | [M-H]^-^ |
| Cytosine | C4 H5 N3 O | 111.0434 | 3.1 | 150685556.7 | [M+H]^+^ |
| 2-Hydroxy-4-methoxybenzaldehyde | C8 H8 O3 | 152.0475 | 26.7 | 144865172 | [M-H]^-^ |
| Polydatin | C20 H22 O8 | 390.1317 | 25.9 | 119932709.8 | [M-H]^-^ |
| Salicylic acid | C7 H6 O3 | 138.0317 | 24 | 114129771.2 | [M-H]^-^ |
| Eriodictyol | C15 H12 O6 | 288.0635 | 30.9 | 111321915.1 | [M-H]^-^ |
| Hydroxygenkwanin | C16 H12 O6 | 300.0635 | 30.8 | 106827252.8 | [M-H]^-^ |
| Azelaic acid | C9 H16 O4 | 188.105 | 28.7 | 98386616.09 | [M-H]^-^ |
| Naringenin | C15 H12 O5 | 272.0686 | 33 | 94665220.56 | [M-H]^-^ |
| Diosmetin | C16 H12 O6 | 300.0636 | 30.6 | 93693743.23 | [M-H]^-^ |
| Astragalin | C21 H20 O11 | 448.1009 | 28.5 | 92589148.98 | [M-H]^-^ |
| Linolenic acid ethyl ester | C20 H34 O2 | 306.2559 | 50.6 | 90722159.79 | [M+H]^+^ |
| Wedelolactone | C16 H10 O7 | 314.043 | 35.8 | 90125731.01 | [M-H]- |
| Glycitein | C16 H12 O5 | 284.0686 | 32.5 | 85220453.16 | [M+H]^+^ |
| Phloridzin | C21 H24 O10 | 436.1371 | 28.8 | 70090171.71 | [M-H]^-^ |
| 5,7-Dihydroxychromone | C9 H6 O 4 | 178.0267 | 28.5 | 62722064.9 | [M-H]^-^ |
| Perillene | C10 H14 O | 150.1047 | 24.3 | 60921856.18 | [M+H]^+^ |
| Caffeic acid | C9 H8 O4 | 180.0424 | 25.1 | 60506125.39 | [M-H]^-^ |
| Ethyl gallate | C9 H10 O5 | 198.0528 | 26.6 | 58865616.08 | [M-H]^-^ |
| Phloretin | C15 H14 O5 | 274.0842 | 28.8 | 58542990.99 | [M+H]^+^ |
| Sibiricose A5 | C22 H30 O14 | 518.1637 | 24.3 | 52749665.86 | [M-H]- |
| Ethylparaben | C9 H10 O3 | 166.0633 | 40.9 | 52105141.57 | [M+H]^+^ |
| Resveratrol | C14 H12 O3 | 228.0789 | 25.9 | 51662996.32 | [M+H]^+^ |
| L-Tryptophan | C30 H48 O | 204.0901 | 23.5 | 50695074.55 | [M-H]^-^ |
| Lupenone | C30 H48 O | 424.3708 | 47.3 | 48772669.64 | [M+H]^+^ |
| Prunetin | C16 H12 O5 | 284.0686 | 34.2 | 47983956.2 | [M+H]^+^ |
| Stachyose | C24 H42 O21 | 666.2229 | 4.7 | 47721854.28 | [M-H]- |
| Antrapurol | C14 H8 O4 | 240.0419 | 27 | 42833504.59 | [M-H]- |
| Ferulaldehyde | C10 H10 O3 | 196.0738 | 30.1 | 42142337.79 | [M+H]^+^ |
| Kaempferol | C15 H10 O6 | 286.0479 | 27.9 | 38978390.85 | [M+H]^+^ |
| Maleic acid | C4 H4 O4 | 116.0109 | 4.3 | 36033468.65 | [M-H]^-^ |
| Isoalantolactone | C15 H20 O2 | 232.1467 | 31.2 | 35085221.51 | [M-H]^-^ |
| Taxifolin | C15 H12 O7 | 304.0585 | 28.6 | 34964456.52 | [M-H]^-^ |
| Parthenolide | C15 H20 O3 | 248.1416 | 40 | 31882793.59 | [M+H]^+^ |
| Methyl gallate | C8 H8 O5 | 184.0372 | 24.4 | 30536187 | [M-H]^-^ |
| Abscisic acid | C15 H20 O4 | 264.1365 | 31.6 | 29023085.72 | [M-H]^-^ |
| Morin | C15 H10 O7 | 302.0426 | 31.4 | 28984753.43 | [M+H]^+^ |
| α-Linolenic acid | C18 H30 O2 | 278.2249 | 47.7 | 25931159.61 | [M+H]^+^ |
| (R)-Mandelic acid | C8 H8 O3 | 152.0473 | 30.9 | 21366167.03 | [M+H]^+^ |
| Methyl hexadecanoate | C17 H34 O2 | 316.2614 | 42.6 | 19928894.94 | [M-H]^-^ |
| Procyanidin B2 | C30 H26 O12 | 578.1431 | 24.2 | 19723657.8 | [M-H]^-^ |
| Cinnamaldehyde | C9 H8 O | 132.0577 | 31.7 | 19160458.2 | [M+H]^+^ |
| Kaempferol-7-O-β-D- glucopyranoside | C21 H20 O11 | 448.1009 | 26.6 | 18816724.15 | [M-H]^-^ |
| Oroxin B | C27 H30 O15 | 594.1591 | 30.5 | 16142717.47 | [M-H]^-^ |
| Trilobatin | C21 H24 O10 | 436.1371 | 25.2 | 16037882.13 | [M-H]^-^ |
| Sinapic acid | C11 H12 O5 | 224.0685 | 27.4 | 15579722.21 | [M-H]^-^ |
| Genistein | C15 H10 O5 | 270.0531 | 30 | 13914508.54 | [M+H]^+^ |
| Ligustilide | C12 H14 O2 | 190.0996 | 26.1 | 12029096.49 | [M-H]^-^ |
| 4-Methoxyphenylacet ic acid | C9 H10 O3 | 166.0631 | 29.6 | 11250983.15 | [M-H]^-^ |
| 6''-O-Acetylglycitin | C24 H24 O11 | 488.1324 | 30.2 | 10147379.88 | [M-H]^-^ |
| 6-Gingerol | C17 H26 O4 | 294.1833 | 38.2 | 8115436.724 | [M-H]^-^ |
