# Supplementary material for: Shengjiang San alleviated sepsis-induced lung injury through its bidirectional regulatory effect
Source: Chin Med. 2023 Apr 17;18:39. doi: 10.1186/s13020-023-00744-6 (PMC10108513; doi:10.1186/s13020-023-00744-6)
Supplement: Supplementary file 7 — Additional file 7: Table S4. 23 Active Compounds. [file 13020_2023_744_MOESM7_ESM.docx]

**Supplementary table 4. 23 Active Compounds**

| **Active compound** | **OB（%）** | **DL** | **Five Principles of drug class** |
| --- | --- | --- | --- |
| Gallic acid | 31.69 | 0.04 | Follow |
| Rheic acid | 47.07 | 0.28 |  |
| Emodin | 24.4 | 0.24 | Follow |
| Purpurin | 18.41 | 0.21 | Follow |
| Cianidanol | 54.83 | 0.24 |  |
| demethylwedelolactone | 72.13 | 0.43 |  |
| Epicatechin | 28.93 | 0.24 | Follow |
| p-Hydroxybenzaldehyde | 29.98 | 0.02 | Follow |
| Eriodictyol | 71.79 | 0.24 |  |
| Hydroxygenkwanin | 36.47 | 0.27 |  |
| Diosmetin | 31.14 | 0.27 |  |
| Linolenic acid ethyl ester | 46.1 | 0.2 |  |
| wedelolactone | 49.6 | 0.48 |  |
| glycitein | 50.48 | 0.24 |  |
| Kaempferol | 41.88 | 0.24 |  |
| Taxifolin | 57.84 | 0.27 |  |
| Morin | 46.23 | 0.27 |  |
| Curcumin | 5.15 | 0.41 | Follow |
| Curcumenol | 91.78 | 0.13 | Follow |
| Demethoxycurcumin | 4.37 | 0.33 | Follow |
| Naringenin | \ | \ | Follow |
| 6-Gingerol | 35.64 | 0.16 |  |
| curcumin | 4.37 | 0.41 | Follow |
